# Stress Appraisal, Stress Mindset, and Perceived Pain During a Cold Pressor Test

**DOI:** 10.1007/s12529-024-10346-7

**Published:** 2025-01-09

**Authors:** Helen Wing Yuk Tse, Marjolein M. Hanssen, Linda M. G. Vancleef, Madelon L. Peters

**Affiliations:** Department of Clinical Psychological Science, Maastricht University, Maastricht, The Netherlands

**Keywords:** Stress appraisal, Stress mindset, Mindset manipulation, Cold pressor test, Speech preparation task

## Abstract

**Background:**

Previous studies demonstrated that task-specific stress appraisals as well as the more general belief that stress is (mal)adaptive (i.e., stress mindset) can affect the stress response. Little is known about the influence of stress appraisals and stress mindset on pain perception. The current study investigated whether stress appraisals and/or stress mindset moderates the impact of stress on pain perception.

**Method:**

Sixty participants performed a stress-inducing speech preparation task followed by the cold pressor test (CPT) to induce pain. Threat appraisal of the speech task was measured with a questionnaire. Stress mindset was manipulated with a video clip emphasizing either the debilitating or enhancing nature of stress, after which another administration of the CPT took place.

**Results:**

Participants in the “stress-is-enhancing” condition reported less pain on the second CPT than on the first, while participants in the “stress-is-debilitating” condition demonstrated similar pain levels. There was no effect of threat appraisals of the speech task on pain perception.

**Conclusion:**

These findings provide evidence on the impact of stress mindset on pain perception. Future studies could extend these findings to patients with pain and examine whether mindset interventions can be a useful component in pain management.

## Introduction

Numerous studies have examined the effects of stress on pain processing and responding. Clinical and experimental studies have demonstrated that acute stress can enhance the experience of pain [1, 2]. However, besides pain enhancement, stress-induced pain reduction has also been reported. This has mostly been the case in animal studies [e.g., 3], but some human studies showing reduced pain after stress are also available [e.g., 4]. The inconsistent findings could at least partly depend on an individual’s appraisal of the stressor.

Lazarus and Folkman distinguished between primary appraisal processes (i.e., what does the situation mean to a person) and secondary appraisal processes (i.e., people’s evaluation of their resources to cope with the situation) [5]. Most studies examining the impact of appraisal on pain perception have centered on the appraisal of pain as varying in the level of threat. A meta-analysis showed that appraising pain as more threatening predicted higher pain intensity and reduced pain tolerance of experimental pain stimuli and higher pain intensity and impairments in patients with clinical pain conditions [6]. Interestingly, appraisals of stressors other than pain itself have also been found to be associated with pain perception [7, 8]. Appraising a mental arithmetic task as more threatening was associated with higher pain during the cold pressor test [8; study 3]. Another study found that more negative primary and secondary appraisals of daily stressors were associated with increased symptom reporting in children with abdominal pain [9].

People differ not only in whether they appraise a specific situation in terms of threat, but also in their more general belief that stress is adaptive or maladaptive. This belief has been referred to as “stress mindset” and is distinct from stress appraisal [10]. An individual can view a situation as threatening, but still believe that it will not harm or that (s)he may even benefit from it (e.g., in building toughness). Crum and colleagues [10] distinguished between a “stress-is-enhancing mindset” (i.e., the belief that stress has positive effects on outcomes such as performance, health and well-being or growth) and a “stress-is-debilitating mindset” (i.e., the belief that stress has negative consequences on these different stress-related outcomes). Several studies have provided evidence that the stress mindset that individuals hold may determine their behavioral, emotional, and physiological responses to a stressor [10–13].

Stress mindset has also been related to the experience of pain. Chronic pain patients were found to have a more negative stress mindset than people without chronic pain [14, 15]. Moreover, while a stress-is-enhancing mindset was associated with less physical pain and better psychological well-being, a stress-is-debilitating mindset was associated with more negative physical symptoms and distress in patients with pain [14, 15]. Furthermore, a stress-is-debilitating mindset was found to be cross-sectionally and prospectively associated with increased somatic symptom reporting in non-patient samples [16–18].

Interestingly, stress mindset can be changed through intervention [19]. Inducing a “stress-is-enhancing” mindset has been shown to lead to more adaptive cognitive, emotional and endocrine responses after a standardized stressor [10, 11] and to a prospectively assessed reduction in self-reported negative health symptoms [20]. Because of the link between stress mindset and somatic symptoms, it may be hypothesized that inducing a stress-is-enhancing mindset could lead to less perceived pain compared to a stress-is-debilitating mindset. However, so far, the influence of a stress mindset manipulation on pain has not been examined.

The aim of the present study was twofold. The first aim was to examine whether appraising a non-pain-related stressor as threatening has an influence on the perception of an experimental pain stimulus. We hypothesized that participants appraising the stress task as more threatening would report more pain during a 1-min cold pressor test (CPT) compared to participants appraising the stress task as less threatening.

The second aim related to the influence of a stress mindset manipulation on pain perception. Pain during the 1-min CPT was assessed before and after manipulating mindset in either a “stress-is-enhancing” or “stress-is-debilitating” direction. We hypothesized that participants receiving an enhancing manipulation would report less pain on the second CPT compared to participants in the debilitating condition, controlled for their pain level on the first CPT.

## Methods

### Participants

To determine the necessary number of participants, sample size calculation was performed for our main analysis, i.e., the stress mindset condition effect on pain ratings. With two conditions, a pre- and post-manipulation assessment and three successive pain ratings for each CPT administration (see below), the basis for our calculation was a 2 × 2 × 3 ANOVA. The first-order condition × time interaction is the effect of interest. Based on previous studies on the effect of manipulated stress appraisal on emotional and physiological outcomes, a medium effect size of d = 0.50 was assumed [11, 21]. With α of 0.05, and power set to 0.90, a minimum of 45 participants were needed.

We recruited a total of 60 bachelor students in psychology (18 men), aged between 17 and 25 years old. They participated in the study in exchange for course credits. Exclusion criteria were pregnancy, cardiovascular or neurological diseases (e.g., epilepsy), Raynaud’s disease, current pain or a past pain disorder, or taking analgesic medications on a regular basis. The criteria were listed in the recruitment poster as well as in the informed consent. The researcher ensured that subjects did not meet the exclusion criteria before they signed the informed consent. The study protocol was approved by the Ethical Review Committee of Psychology and Neuroscience (ERCPN) of Maastricht University.

### Pain Induction

Pain was induced by means of the CPT. Participants immersed their hand for 1 min in a water bath that was maintained at a constant temperature of 5 °C. A Plexiglas tank of 36 × 30 × 15 cm (W × L × D) with an open heating bath circulator and an immersion cooler was used (Julabo ED‐19A; Julabo Seelbach, Germany). Participants could withdraw their hand from the water before the minute was over if they could no longer tolerate the pain. To ensure that participants started with the same initial hand temperature, they first immersed their hand in a tank containing water at 22 °C (1 min).

### Stress Induction and Stress Mindset Manipulation

The stress induction consisted of a speech preparation task. Speech preparation tasks have been used previously to induce stress and were found to lead to physiological and subjective stress responses [22–24]. In our version of this task, participants were told that they had to give a 5-min speech on the topic of “The biology of stress” in front of two research assistants waiting for them in another room. They were given 5 min to prepare for this speech. To further increase their stress level, they were told that the research assistants would score their verbal and nonverbal signs of stress, and the presentation would be videotaped and scored later for their facial expressions. During the preparation period, the researcher left the room. The speech preparation task was done twice, before the first and before second CPT.

Stress mindset was manipulated by showing participants a video clip of approximately 4 min that either emphasized the positive or negative consequences of stress (i.e., the enhancing and debilitating condition). In the enhancing condition, participants watched an excerpt of the TED Talk “How to make stress your friend” (Kelly McGonigal: https://www.youtube.com/watch?v=2R83PyzXadQ&feature=youtu.be%E2%80%8B [0:00–4:46]). In this video, a Stanford health Psychologist explains that the way you think about stress can transform your experience of it, and that stress not necessarily has negative consequences for health. In the debilitating condition, participants watched the TED-Ed talk “How stress affects your body” (Sharon Horesh Bergquist: https://www.youtube.com/watch?v=v-t1Z5-oPtU [0:00–4:07]). This video explains that stress leads to the release of endocrine and immune factors, which may cause high blood pressure, may damage to cells and organs, and ultimately may lead to chronic diseases.

### Measures

#### Perceived Pain

Participants verbally scored perceived pain during the CPT by looking at the “Pain Thermometer Scale” which ranged from 0 to 100 (i.e., 0 = no pain and 100 = extreme pain). The researcher prompted pain intensity ratings at 20, 40, and 60 s after initiating hand immersion. If pain tolerance was reached before the 60-s immersion time, missing values were replaced by the value 100, indicating maximum pain sensitivity.

#### Perceived Stress, Stress Mindset, and Appraisal of the Speech Task

Perceived stress was measured at four time points, i.e., at the beginning of the experiment, after the first speech preparation task, after the stress mindset manipulation, and after the second speech preparation task. We used a single question: How stressed are you right now? The item was scored on a 7-point Likert scale, ranging from 1 (extremely stressed) to 7 (extremely relaxed). To facilitate interpretation, scores were reversed for the analyses, such that lower scores denote less stress. Stress mindset was assessed twice, before and after the mindset manipulation, with the question “How do you perceive stress?” The item was scored on a 5-point Likert scale, with the labels 1 (very negative, it can be harmful to your physical and mental health) and 5 (very positive, it allows you to grow as a person).

Appraisal of the speech task was measured after both preparation phases with the Primary Appraisal Secondary Appraisal (PASA) questionnaire [25]. The PASA has two primary appraisals subscales, i.e., “Threat” and “Challenge.” Both subscales consist of four items that are measured on a 6-point Likert scale ranging from 1 (I completely disagree) to 6 (I completely agree). Cronbach’s alphas for the threat scale were satisfactory, 0.84 and 0.83 for the first and second assessments. Cronbach’s alphas for the challenge scales were insufficient, i.e., 0.62 and 0.64 for the first and second assessments. Because Cronbach’s alpha for the challenge scale is insufficient and challenge does not form part of the hypotheses, this scale was not used in the analyses.

#### Pain Catastrophizing and Appraisal of the CPT

Pain catastrophizing and appraisal of the CPT were included in the analyses as potential covariates affecting pain perception. Pain catastrophizing was measured with the Pain Catastrophizing Scale (PCS; [26]). The PCS consists of 13 items that are scored on a 5-point Likert scale (1 = not at all; 5 = all the time). Cronbach’s alpha in the current study was 0.92. Appraisal of the CPT was measured with the PASA scale as above, but now the questions referred to appraisal of the CPT. Cronbach’s alphas were 0.80 and 0.89 for the first and second assessments.

### Procedure

After signing informed consent, participants filled out the PCS, and the items assessing stress mindset and perceived stress at baseline. Next, the stress induction took place. Participants were instructed to prepare a 5-min speech on “the biology of stress,” during which the experimenter left the room. After 5 min, the experimenter returned and the first CPT was administered. Participants verbally scored their pain scores at 20, 40, and 60 s after initiating hand immersion.

**Fig. 1:**
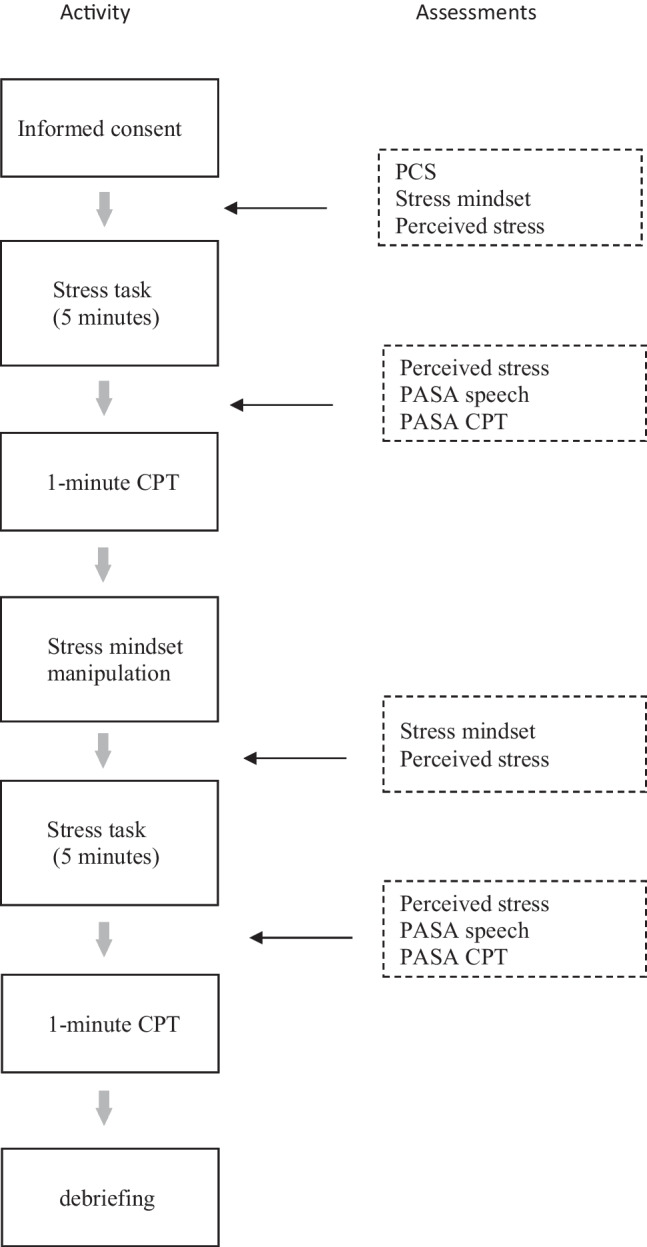
Overview of the procedure

After this first pain induction, the mindset manipulation took place. Participants were randomized by the online survey software Qualtrics to receive either the stress-is-enhancing or the stress-is-debilitating video clip. To increase attention to the content of the clip, participants were told that they could use the information in the video in their presentation. After the video, another 5 min followed in which participants could prepare their presentation. The second CPT was administered thereafter. Pain scores during CPT2 were again given at 20, 40, and 60 s. This concluded the experimental session. Participants were informed that they did not need to give the presentation and were debriefed about the study’s purpose. Figure 1 gives an overview of the procedure and the timing of the assessments.

### Statistical Analysis

The effectiveness of the stress induction was examined by means of an ANOVA for repeated measures with the four perceived stress scores as dependent variable. The time effect (baseline versus the three scores post stress induction) was the effect of interest. The effect of the mindset manipulation was tested with a 2 × 2 ANOVA for repeated measures with time (before vs after the manipulation) as a within-subject factor and condition (enhancing vs debilitating) as a between-subject factor.

To examine the effect of threat appraisal of the speech task on perceived pain during the first CPT (i.e., before the mindset manipulation), a linear regression analysis was performed. A total pain score was calculated by summing the pain ratings at 20, 40, and 60 s immersion time. Next, this total pain score was regressed on threat appraisal of the speech task, threat appraisal of the CPT, pain catastrophizing, and sex. Threat appraisal of the speech task was the predictor of interest. Sex, pain catastrophizing, and threat appraisal of the CPT were included as control variables because they may affect pain scores when using the CPT [26–28]. For the second CPT, a similar regression analysis was performed but now with the threat appraisal scores at post-manipulation as predictors. Because the second CPT took place after the stress mindset manipulation, the potential effect of the condition and the condition × threat appraisal interactions on pain intensity was also examined.

The influence of stress mindset on pain ratings was examined with a 2 × 3 × 2 ANOVA for repeated measure with time (before vs after appraisal induction) and immersion duration (20 s, 40 s, 60 s) as within-subject factors, condition (debilitating vs enhancing) as between-subject factor, and sex and pain catastrophizing as covariates. Effect sizes are reported as partial eta squared (η_p_^2^) for the ANOVA and as Cohen’s d for post hoc t-tests, where 0.01, 0.06, and 0.14 (η_p_^2^) and 0.2. 0.5, and 0.8 (Cohen’s d) are taken as indicating small, medium, and large effect sizes, respectively [29].

## Results

### Descriptive Analyses

In the enhancing condition, there were seven men, in the debilitating condition 11. Age was queried using categories (17–20; 21–25; > 25). In the enhancing condition, 19 participants and, in the debilitating condition, 17 participants fell in the 21–25-year category. The remaining participants were between 17 and 20 years old. Pain catastrophizing, perceived stress, stress mindset, and threat appraisals did not significantly differ between the groups at baseline. Means and standard deviations for baseline variables and their correlations are displayed in Table 1. Participants reported to be moderately stressed at baseline (*M* = 3.82; *SD* = 1.48) and generally perceived stress as negative (*M* = 2.20; *SD* = 0.86).

**Table 1:** Baseline descriptives and Pearson correlations

	Enhancing condition	Debilitating condition	Total	Perceived stress		Stress mindset	Threat speech	Threat CPT
	Mean (SD)	Mean (SD)	Mean (SD)	Pearson correlations				
PCS	31.90 (11.28)	30.50 (8.85)	31.20 (10.08)	.19	.29*		.33**	.40**
Perceived stress	4.07 (1.48)	4.30 (1.49)	4.18 (1.49)		.34**		.34**	.03
Stress mindset	2.30 (1.02)	2.10 (0.66)	2.20 (0.86)				.55**	.11
Threat speech	13.80 (4.51)	13.40 (3.63)	13.60 (4.06)					.22
Threat CPT	10.40 (3.45)	11.13 (4.03)	10.77 (3.74)					

*PCS*, pain catastrophizing scale; *Threat speech*, threat appraisal of speech task; *Threat CPT*, threat appraisal of cold pressor test

Note that lower scores of stress mindset denote a more negative mindset

**Correlation is significant at the 0.01 level (2-tailed)

*Correlation is significant at the 0.05 level (2-tailed)

Participants with higher pain catastrophizing scores had a more debilitating stress mindset and appraised the speech task and especially the CPT as more threatening (Table 1). Participants with a more debilitating stress mindset scored higher on perceived stress at baseline and appraised the speech task as more threatening. Threat appraisal of the speech task was unrelated to threat appraisal of the CPT.

Four participants (all female; three in the enhancing condition) withdrew their hand before the limit of 60 s during the first CPT (three participants between 20 and 40 s, one between 40 and 60 s). During the second CPT, three of them again withdrew their hand prematurely (two in enhancing condition). No additional participants withdrew prematurely during the second CPT. Pain ratings for the missing time points were replaced by 100 to indicate intolerable pain.

### Manipulation Checks

A repeated measures ANOVA showed a significant time effect for perceived stress (*F*(3.56) = 14.75, *p* < 0.01; η_p_^2^ = 0.44). Perceived stress was significantly higher after the first speech preparation phase (*M* = 5.17, *SD* = 1.03; *t*(59) = 6.00, *p* < 0.01; d = 0.76), after the stress mindset manipulation (*M* = 4.57, *SD* = 1.06; *t*(59) = 2.06, *p* = 0.42; d = 0.27), and after the second speech preparation phase (*M* = 4.83, *SD* = 1.06; *t*(59) = 3.23, *p* = 0.02; d = 0.42) than at baseline (*M* = 4.18, *SD* = 1.48). There was no time × condition interaction; thus, perceived stress was not affected by the mindset manipulation.

Table 2 shows the scores for stress mindset before and after the mindset manipulation in the two conditions. Whereas after the enhancing video stress mindset became more positive, after the debilitating video, mindset became more negative. The Time × Condition interaction reached significance (*F*(1, 58) = 23.77, *p* < 0.01; η_p_2 = 0.29) indicating that the video clips had the intended effect. The stress mindset manipulation did not affect threat appraisals, neither for the speech task nor for the CPT (Table 2).

**Table 2:** Mean and SD for stress mindset and perceived stress before and after the mindset manipulation

	Enhancing condition Mean (SD)		Debilitating condition Mean (SD)	
	Pre	Post	Pre	Post
Stress mindset	2.30 (1.02)	3.00 (0.98)	2.10 (0.66)	1.80 (0.76)
Threat speech	13.80 (4.51)	13.37 (3.83)	13.40 (3.63)	13.20 (3.94)
Threat CPT	10.40 (3.45)	14.57 (4.77)	11.13 (4.03)	13.87 (4.86)
Pain at 20 s	50.9	47.5	40.4	41.4
Pain at 40 s	64.2	59.0	57.7	59.3
Pain at 60 s	69.2	65.2	67.7	65.3

*Threat speech*, threat appraisal of speech task; *Threat CPT*, threat appraisal of cold pressor test. Higher scores on stress mindset denote a more enhancing mindset

### Threat Appraisals and Perceived Pain

At baseline, participants perceived the speech task as more threatening than the CPT. Threat appraisal of the CPT increased after the first administration with the second CPT appraised as significantly more threatening than the first CPT (*M* = 10.77; *SD* = 3.74 vs *M* = 14.22; *SD* = 4.79; *t*(59) = 6.71, *p* < 0.01; d = 0.87). Threat appraisal of the speech task was similar for the first and second assessments (*M* = 13.60; *SD* = 4.06 vs *M* = 13.28; *SD* = 3.85, ns.).

The result of the regression model predicting pain intensity during the two CPTs is displayed in Table 3. Appraising the speech task as more threatening did not affect pain ratings in either of the two CPTs. Pain catastrophizing significantly predicted perceived pain during the first CPT, while both pain catastrophizing and threat appraisal regarding the CPT significantly predicted pain intensity during the second CPT. To control for a potential influence of the stress mindset manipulation on pain during the second CPT, the main effect of condition and the interaction condition × threat appraisal of the speech task were added to the model. Neither of these two additional predictors contributed significantly to the model.

**Table 3:** Regression analyses with threat appraisals as predictor of perceived pain intensity during the first and second CPT

Predictor variable	B (95% CI)	Beta	t (p)
*Perceived pain intensity CPT1*			
Sex	− 4.33 (− 45.33 to 36.66)	− 0.03	− 0.22 (.83)
PCS	**1.98 (0.15 to 3.81)**	**0.31**	**2.17 (.04)**
Threat speech	2.15 (− 2.33 to 6.63)	0.14	0.96 (.34)
Threat CPT	0.49 (− 4.46 to 5.44)	0.03	0.20 (.84)
Model summary	*R*^2^ = 0.14, *F*(5,54) = 2.26, *p* = .08		
*Perceived pain intensity CPT2*			
Sex	− 12.42 (− 43.45 to 18.61)	− 0.09	− 0.80 (.43)
PCS	**1.62 (0.16 to 3.08)**	**0.25**	**2.22 (.03)**
Threat speech	0.79 (− 2.98 to 4.56)	0.05	0.42 (.68)
Threat CPT	**7.22 (4.10 to 10.33)**	**0.54**	**4.64 (.00)**
Model summary	*R*^2^ = 0.45, *F*(5,54) = 11.42, *p* < .01		

*PCS*, pain catastrophizing scale; *Threat speech*, threat appraisal of speech task; *Threat CPT*, threat appraisal of cold pressor test. CPT1: summed pain scores over 20, 40, and 60 s for first CPT; CPT2: summed pain scores over 20, 40, and 60 s for second CPT. Significant regression results in bold. B is the unstandardized coefficient; Beta is the standardized coefficient

### Stress Mindset and Perceived Pain

**Fig. 2:**
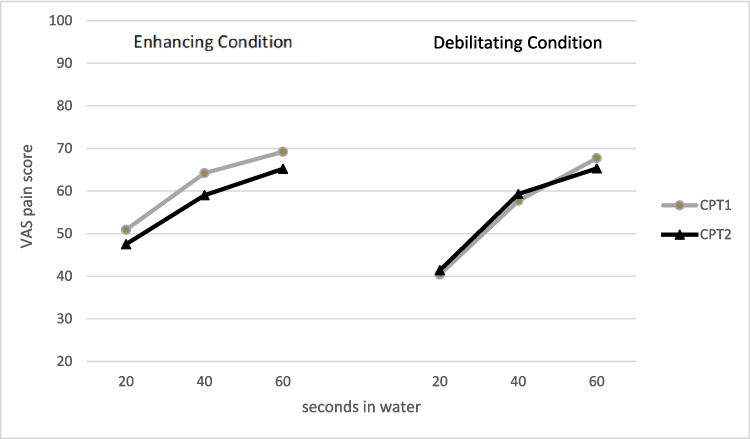
VAS pain scores (0–100) during the first and second cold pressor tests assessed at 20, 40, and 60 s after immersion in the two conditions

Table 2 and Fig. 2 show the pain scores at 20, 40, and 60 s for the first and second CPT per condition. In the enhancing condition, participants reported less pain during the second CPT at all three time points, whereas there was hardly any change in pain reports in the debilitating condition. The time (before vs after mindset manipulation) × condition (debilitating vs enhancing) effect on CPT pain scores did not reach significance (*F*(1,56) = 3.32, *p* = 0.07; η_p_^2^ = 0.06), but the time × immersion duration x condition did (*F*(2,55) = 4.12, *p* = 0.02; η_p_^2^ = 0.13). Post hoc analyses within conditions showed that within the enhancing condition pain intensity was lower during the second CPT compared to the first at 40 s (*t* = 2.23, *p* = 0.03; d = 0.41) and at 60 s (*t* = 2.24, *p* = 0.03; d = 0.41). Although pain ratings did not differ at 20 s, the overall time effect in the enhancing condition was significant (*F*(1,29) = 4.45, *p* = 0.04; η_p_^2^ = 0.13). In the debilitating condition, there were no differences between pain reports between the first and second CPT at any of the time points, and hence, there was no significant time effect (*F*(1,29) < 0.01, *p* = 0.98; η_p_^2^ = 0.00).

## Discussion

This study examined whether the appraisals of a non-pain-related stressor and/or stress mindset influence perceived pain intensity. The main results can be summarized as follows. Threat appraisal of the stress task did not affect perceived pain, while stress mindset did. Inducing a stress-is-enhancing mindset led to lower perceived pain during the post-manipulation CPT compared to the pre-manipulation CPT. Inducing a stress-is-debilitating mindset did not lead to a change in perceived pain.

Our first hypothesis, which assumed that participants who appraised the speech preparation task as more threatening would perceive more pain in a subsequent CPT, was not supported. Threat appraisal of the CPT did show a significant association with perceived pain, but during the second CPT only. Moreover, pain catastrophizing, which can be viewed as a stable and generalized negative appraisal of pain, was associated with perceived pain during both CPT administrations. The influence of such pain-relevant threat appraisals on perceived pain is in line with many previous studies [26, 28].

The second hypothesis stated that manipulating stress mindset would have an effect on pain during the subsequent CPT. The manipulation check indicated that the videos were effective in inducing the intended mindset; a large effect size was found for the condition effect for the pre- to post-manipulation change in stress mindset. As hypothesized, participants in the stress-is-enhancing condition reported less pain during the second CPT compared to the first, while those in the stress-is-debilitating condition reported similar pain levels during both CPTs. The difference between conditions showed a moderate to large effect size, but was only apparent on the two latter time points (i.e., 40 and 60 s) during the CPT. This concurs with the findings of other manipulations affecting CPT pain that have similarly shown that especially later time points, when the pain becomes more intense, are affected [e.g., 30].

As far as we are aware, the current study is the first to examine the manipulation of stress mindset in the context of pain. Previous studies have found that a stress-is-disabling mindset was associated with increased disability in patients with chronic pain while a stress-is-enhancing mindset was associated with higher well-being [14, 15]. In these studies, patients’ habitual mindset was assessed and no mindset intervention was given. A recent study manipulated mindset regarding the side effects of the COVID-19 vaccine and reported that participants receiving a positive manipulation (i.e., information that side effects demonstrate the vaccine is working) reported fewer side effects directly after vaccination [31]. These side effects included muscle aches and pain at the side of injection, among others. Here we demonstrated that a positive stress mindset can affect the experience of pain in an experimental context.

The stress mindset manipulation did not affect the perceived stressfulness of the speech task, nor the threat appraisal of either the speech or the pain task. This concurs with the notion that stress mindset is a meta-cognitive belief about stress in general and is independent of the amount of stress one experiences or the appraisal of that stress [11]. Inducing a stress-is-enhancing mindset will therefore not necessarily lead to experiencing less stress, but rather to a more beneficial reaction to stress [10, 11, 21]. Other benefits of a stress-is-enhancing mindset were less anxiety and depression, improved work performance, decreased attentional bias to negative material, better self-reported health, and even lower mortality [10, 20, 21, 32]. The current study suggests that these benefits can be extended towards less sensitivity for pain.

Future studies may examine whether stress mindset interventions can be helpful for patients with chronic pain to alleviate pain and/or the impact that pain has on well-being and functioning. Grunenwald and colleagues [15] suggested that a mindset module could be included in pain therapy, alongside other proven effective components. However, one should carefully consider the format such a module should take. While the current mindset intervention is well-suited within an experimental context, its effects may be short-lasting, and less appropriate for use in a clinical setting.

Recently, Crum and colleagues proposed a more sustainable intervention, taking into account that people may encounter contradicting evidence regarding the role of stress [20]. This so-called meta-cognitive approach teaches people what a mindset is and how it can affect outcomes. Balanced information about stress is given and participants are told that even though a positive stress mindset is not necessarily true, adopting such a mindset may have beneficial effects. In three successive experiments, Crum et al. demonstrated that this approach can be an effective and sustainable method to induce a more positive stress mindset and that it led to less health complaints and better performance in a work setting [20]. One may propose that such a meta-cognitive mindset intervention could be well-suited for patients with chronic pain.

The present study has several limitations. First, the videos that were used to induce an enhancing versus a debilitating mindset were not matched for type of images, colors, and sounds. Although videos had the intended effect, i.e., changing mindset in the desired direction, it is recommended that future studies use videos that are more similar in look and feel, such as those created by Crum and colleagues [10].

Second, we did not include a no-intervention control group, and therefore, we cannot conclude whether the enhancing mindset led to decreased pain sensitivity, or the debilitating mindset to increased pain sensitivity. The decreased pain rating in the enhancing condition may have been due to the effect of the manipulation or because of repeated exposure of the CPT. In the latter case, it would rather be the debilitating condition counteracting this habituation effect. It may be noted, however, that at baseline participants had a predominantly debilitating mindset, and therefore changing the mindset towards “enhancement” may have had a larger impact than reinforcing the already dominant mindset. Our results show that the change in mindset was indeed the largest after the enhancing condition, aligning with earlier findings [10].

Similarly, we did not have a no stress control group. The speech preparation task was meant to induce anticipatory stress, with the idea that participants appraising this task as more threatening would show a larger impact of the stressor on their pain perception. Our results show that the speech preparation task indeed resulted in a significant increase in perceived stress, with a large effect size, and was appraised as moderately threatening. However, whether or not this stress influenced pain perception cannot be established. We also do not know whether the effect of the stress mindset manipulation on perceived pain would have worked without the prior stressor. Previous experimental studies using film clips showed that stress mindset can be changed also in the absence of a stressor.

Another limitation is that we assessed mindset with a single question instead of the 8-item Stress Mindset Measure [SMM; 10]. This more elaborate questionnaire assesses multiple perceived beneficial or detrimental effects of stress and may yield a more complete picture of the effect of the intervention. Additionally, threat appraisal of the CPT was measured by means of the PASA, while this scale has originally been developed and validated for use with the trier social stress task [TSST; 33]. Although the content of the items can relate to different stressors, it has not formally been validated for use in this context.

In conclusion, the present study showed that stress mindsets can have an impact on perceived pain. Together with findings from earlier studies showing that patients with pain-related disorders generally have a more negative stress mindset compared to individuals without pain, this suggests that stress mindset interventions could have a role in pain management. Further studies in clinical populations are warranted.

## Data Availability

The data that support the findings of this study are openly available in dataverseNL at https://doi.org/10.34894/XI8PO0
